# Supplementary material for: Gel phase formation in dilute triblock copolyelectrolyte complexes
Source: Nat Commun. 2017 Feb 23;8:14131. doi: 10.1038/ncomms14131 (PMC5331217; doi:10.1038/ncomms14131)
Supplement: Supplementary Information — Supplementary Figures, Supplementary Methods and Supplementary References [file ncomms14131-s1.pdf]

Supplementary Figures

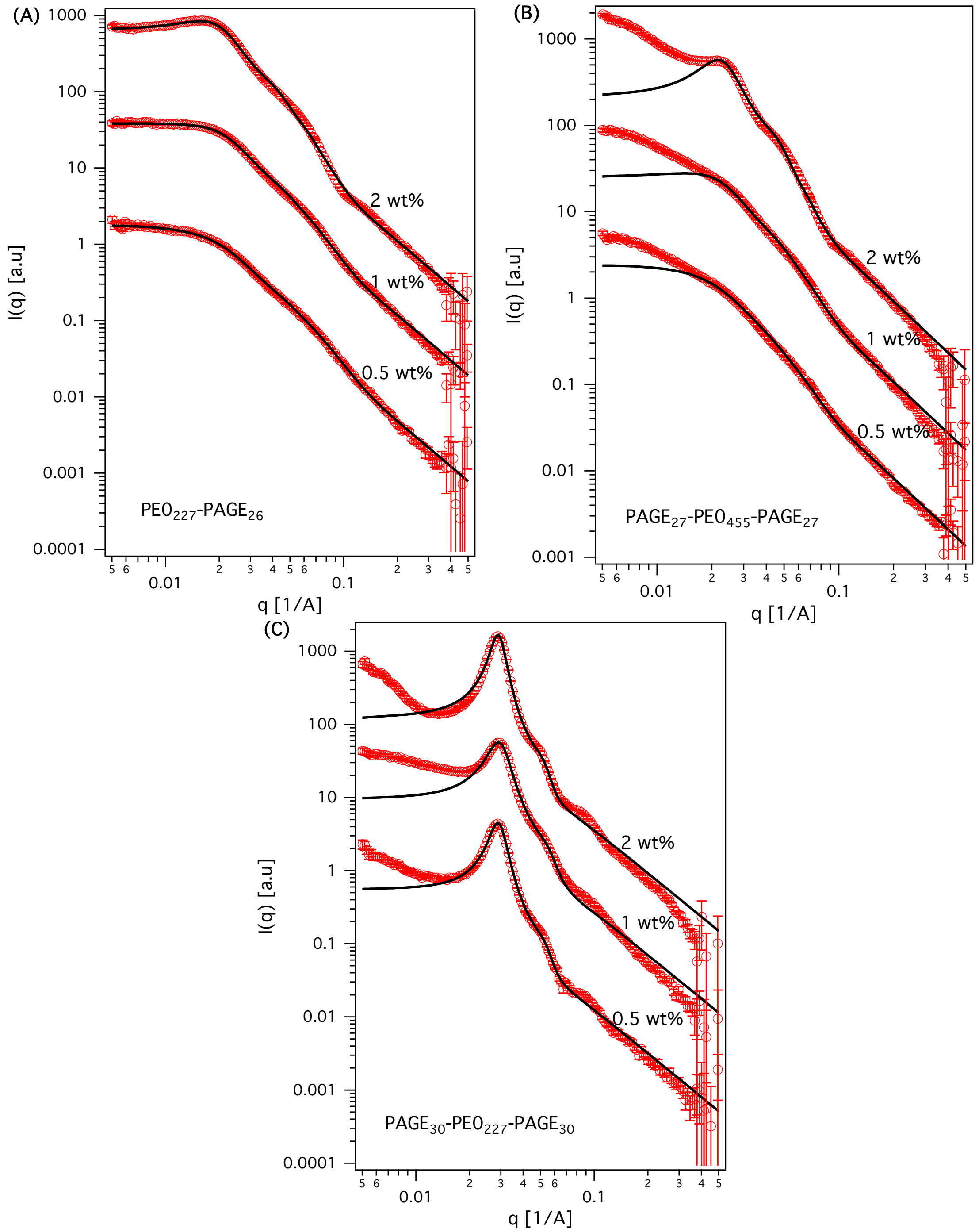

Supplementary Figure 1: SANS scattering intensities and the model fits for various oppositely charged di- and triblock copolyelectrolyte assemblies.

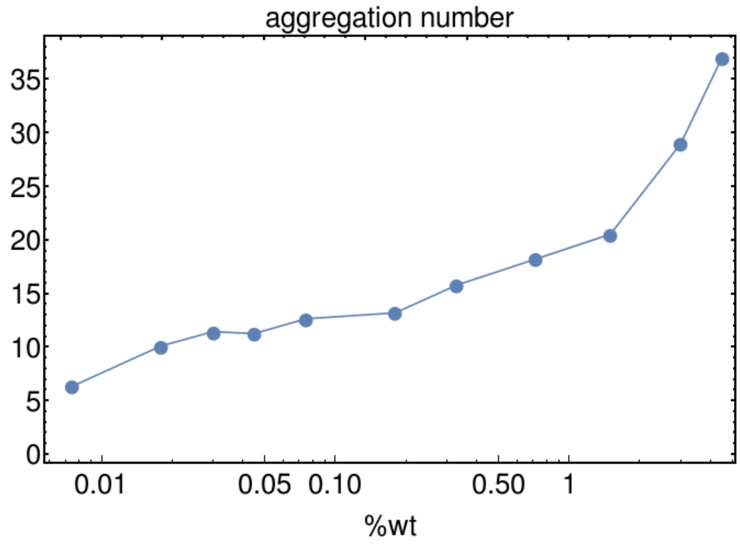

Supplementary Figure 2: Chain aggregation number for the PEC domains in triblock copolyelectrolyte assemblies with varying polymer concentrations. The aggregation number increases initially with polymer concertation before achieving a plateau around 0.04 wt%. The growth at higher polymer is expected to be an artefact resulting from the implicit solvent conditions in the simulations.

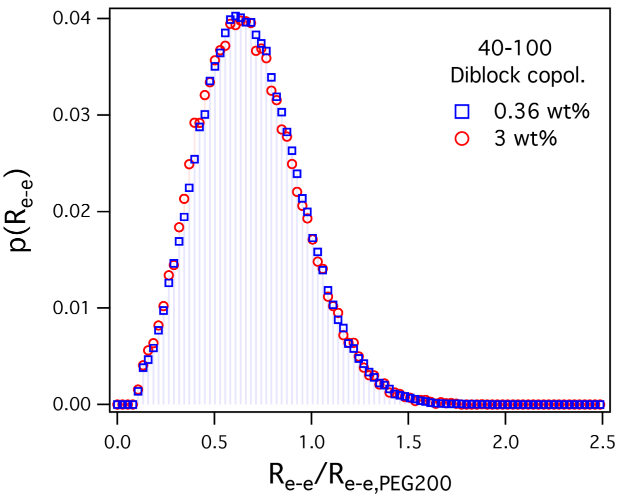

Supplementary Figure 3: Distribution of the end-to-end distances of the neutral block in the diblock copolyelectrolyte micelles at $\phi = 0.36$ and 3 wt%.

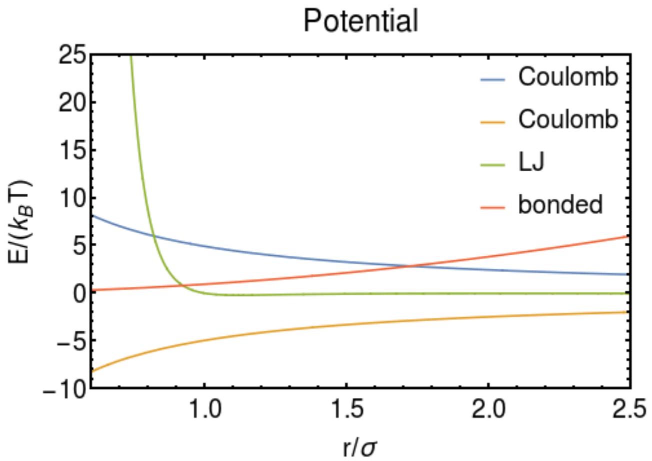

Supplementary Figure 4: Potentials employed in the simulations. Shown Coulomb potential for same charge particles -repulsive and same charge attractive. Lennard-Jones potential -repulsive and harmonic bonded potential.

Supplementary Methods

Analysis of the SANS scattering data

The SANS scattering profiles were fit via a least-squares minimization in MATLAB using the models for PEC micelle scattering described previously in the literature[1]. The experimental data and the corresponding fits are shown in Supplementary Fig. 1 for the diblock and triblock copolyelectrolyte assemblies.

Supplementary References

1. Krogstad, D. V. *et al.* Small Angle Neutron Scattering Study of Complex Coacervate Micelles and Hydrogels Formed from Ionic Diblock and Triblock Copolymers. *J. Phys. Chem. B* **118,** 13011–13018 (2014).
